# Investigating the effect of increased dopamine signaling on cerebral blood flow in Major Depressive Disorder: a double-blind randomized placebo-controlled study

**DOI:** 10.1038/s41386-026-02471-6

**Published:** 2026-06-26

**Authors:** Matti Gärtner, Marvin S. Meiering, David Weigner, Moritz Hempel, Rebecca Gruzman, Luisa Carstens, Christian Keicher, Maarten Mennes, Christian F. Beckmann, Gerd Luippold, Sigurd D. Süssmuth, Margot Popp, Andreas Wunder, Simone Grimm

**Affiliations:** 1 MSB Medical School Berlin, Berlin, Germany; 2 Department of Education and Psychology, Freie Universität Berlin, Berlin, Germany; 3 Charité Research Organisation GmbH, Berlin, Germany; 4 SBGneuro Ltd, Littlemore, Oxford, UK; 5 Donders Institute, Centre for Medical Neuroscience, Radboud University Medical Centre, Nijmegen, Netherlands; 6 Centre for Functional MRI of the Brain (FMRIB), Nuffield Department of Clinical Neurosciences, Wellcome Centre for Integrative Neuroimaging, University of Oxford, Oxford, UK; 7 Clinical Development and Operations, Boehringer Ingelheim Pharma GmbH & Co. KG, Biberach an der Riss, Germany; 8 Medicine Therapeutic Area CNS-Retinopathies-Emerging Areas, Boehringer Ingelheim International GmbH, Biberach an der Riss, Germany; 9 Private Consultant, Lübeck, Germany

**Keywords:** Neuroscience, Depression

## Abstract

Anhedonia, a core symptom of major depressive disorder (MDD), is linked to dysfunction in reward-related brain regions and impaired dopamine (DA) signaling. This study used arterial spin labeling (ASL) to investigate the effects of a single low dose of amisulpride, a selective DA antagonist, on cerebral blood flow (CBF) in MDD patients and healthy volunteers (HVs). In a double-blind, placebo-controlled, randomized, parallel-group design, participants received either 100 mg amisulpride or placebo. *N* = 111 participants (MDD: *n* = 57; HV: *n* = 54) were included in the final analysis and group differences were assessed in core regions of the reward network, particularly the ventral striatum. Individual CBF values were analyzed in relation to self-reported anhedonia and depression severity. Amisulpride was associated with higher perfusion in the ventral striatum compared to placebo in both HVs and MDD patients, consistent with enhanced dopaminergic activity in this region. No significant CBF changes were observed in other areas of the reward network. Ventral striatal perfusion was significantly associated with self-reported anhedonia. Exploratory whole-brain analyses revealed higher CBF in the right frontal operculum in MDD patients compared to HVs under placebo, but not under amisulpride. In the placebo group, both anhedonia and depression severity were associated with CBF in the right frontal operculum; these associations were absent following amisulpride administration. In conclusion, we provide initial evidence of region-specific effects of amisulpride administration on CBF that are consistent with modulation of dopaminergic signaling. Increased perfusion in the ventral striatum suggests a potential dopaminergic mechanism relevant to anhedonia. These findings underscore the ventral striatum’s role in reward processing and support its involvement in the pathophysiology of anhedonia across clinical and non-clinical populations.

## Introduction

Most patients with major depressive disorder (MDD) experience anhedonia [1], a loss of interest or pleasure in nearly all activities (DSM-5 [2]). Anhedonia is a transdiagnostic symptom that is also present in other mental disorders, such as schizophrenia or borderline personality disorder [3], involves distinct reward- and motivation-related processes, and is highly relevant to patients’ daily functioning. Findings from previous studies in MDD indicate that anhedonia cannot be sufficiently treated with antidepressant medication [4–6] and that deficits in reward- and motivation-related processing are key predictors for poor treatment response [7, 8]. Accumulating neuroimaging evidence suggests that alterations in a reward network comprising cortical (e.g., ventromedial prefrontal cortex, anterior cingulate cortex, insula) and striatal regions (e.g., nucleus accumbens, caudate, putamen) [3, 9, 10] associated with lowered dopamine (DA) function [11, 12] are linked to these deficits [13]. Thus, selective pharmacological manipulation of dopamine signaling provides an opportunity to study dysfunction in neural networks involved in the processing of reward and motivation.

Previous studies showed that the selective D2/D3 antagonist amisulpride impacts striatal activation and corticostriatal functional connectivity [14, 15]. At high doses ( ≥ 400 mg), amisulpride is an effective neuroleptic, as shown by its efficacy in ameliorating positive symptoms of psychosis [16]. At low doses ( ≤ 200 mg), it has demonstrated antidepressant properties [17–19]. These qualitatively different functional outcomes of low and high doses might be attributed to dose-dependent differences in D2/D3 receptor occupancy, with lower doses associated with relatively greater influence on presynaptic autoreceptors and higher doses leading to substantial postsynaptic receptor blockade [20]. Importantly, this transition is not discrete but reflects a gradual shift in the balance of pre- and postsynaptic effects, influenced by receptor affinity, regional distribution, and pharmacokinetic factors. Supporting this dose- dependent framework, studies in healthy individuals have shown that high doses (400 mg) reduce striatal BOLD signal during reward tasks, whereas lower doses enhance reward learning and increase striatal activity [21]. However, results have been inconsistent [22], potentially due to the inherent limitations of BOLD contrast imaging, which provides an indirect and complex measure of neural activity [23, 24].

In contrast, arterial spin labeling (ASL) measures regional cerebral blood flow (rCBF), a single physiological parameter that is temporally stable and relatively straightforward to interpret [25]. Although ASL provides an indirect measure of neural activity via cerebral perfusion, its validity is supported by studies demonstrating convergence with established PET-based techniques [26–29]. While ASL-derived perfusion has mainly been used to investigate cerebrovascular diseases, dementia, and neuro-oncological disorders, it is gaining traction in psychiatry and other fields of neuroscience as a research tool [30]. Using ASL, numerous studies have reported CBF abnormalities in MDD, particularly in prefrontal cortex, middle and inferior frontal cortices, temporal regions, thalamus, anterior cingulate cortex, basal ganglia, and insula [31–45] thus establishing CBF as a potential imaging biomarker for this disorder. However, the directionality of perfusion changes (increased or decreased CBF) is variable between regions and studies, which may be due to limited statistical power from relatively small samples, clinical heterogeneity related to illness profile variation, and methodological differences [46]. Given ASL’s sensitivity for the detection of drug effects [47, 48], it may be ideally suited to study the acute effects of amisulpride on neural activity and may provide insights into its antidepressant mechanism of action [48, 49]. To our knowledge, only one prior study investigated the effect of low dose amisulpride on cerebral perfusion in a small sample of 20 healthy subjects and reported perfusion decreases in prefrontal areas, but increases in the basal ganglia [50].

However, no study to date has investigated how a transient increase in dopamine signaling affects cerebral blood flow in patients with MDD. Therefore, this study aimed to examine the effects of a single low-dose administration of amisulpride—hypothesized to block presynaptic autoreceptors and thereby enhance dopamine signaling—on cerebral perfusion within key regions of the reward network in a large sample of healthy individuals and patients with MDD. We hypothesized that amisulpride administration would be associated with higher cerebral perfusion in core regions of the reward network, particularly the ventral striatum, compared to placebo. Furthermore, we expected that higher perfusion in these regions would be positively associated with self-reported anhedonia and depression severity.

## Methods

### Participants

Male and female healthy volunteers (HV) and patients with major depressive disorder (MDD) aged 18–45 years were recruited by the Charité Research Organisation, a contract research organization and subsidiary of the Charité—Universitätsmedizin Berlin, to take part in the study. A total of *N* = 120 participants completed the study procedures. A detailed overview of participant flow, including screening outcomes and exclusions, is provided in the Supplementary Material (Fig. S1). Main inclusion criteria for the MDD group were a current diagnosis of MDD and a MADRS score between 7 and 25 at screening, indicating mild to moderate symptom severity. Only unmedicated patients were included to avoid potential confounding effects of pharmacological treatment. Additionally, individuals with higher symptom severity were excluded to reduce the likelihood of psychomotor retardation, which is more common in severe depression [51], and may interfere with task-based study outcomes. Patients were excluded if (1) they met diagnostic criteria for any major psychiatric disorder (other than MDD), as determined by a structured clinical interview (SCID-5) led by trained psychologists; (2) they received prescribed medication (including antidepressants) within 14 days or fluoxetine within 90 days prior to the fMRI measurement; (3) they received psychotherapy within 14 days prior to the fMRI measurement. The healthy volunteers (HV) did not have a history of psychiatric illness and completed the same assessment protocol as the MDD group. Individuals within the HV group were excluded if they (1) met diagnostic criteria for any major psychiatric disorder, as determined by SCID-5 at screening; (2) had a lifetime history of psychiatric or neurologic disorders. Details of the complete inclusion and exclusion criteria are described in Carstens et al. [52]. All participants gave written informed consent and received monetary compensation for participation. The study was conducted in compliance with Good Clinical Practices (ICH-GCP), the Declaration of Helsinki, it was approved by the local ethics committee (Landesamt für Gesundheit und Soziales, Ethical Review Board of the State of Berlin, Berlin, Germany), and registered at ClinicalTrials.gov (identifier: NCT05347199).

### Experimental design & procedure

In a double blind, placebo-controlled, randomized, single dose, parallel-group study, we investigated the effects of a single dose of amisulpride on cerebral blood flow (CBF) across four treatment arms (MDD/placebo, MDD/amisulpride, HV/placebo, and HV/amisulpride). Amisulpride exhibits a biphasic plasma concentration profile, with peak levels occurring approximately 1 h and again 3–4 h following oral administration, and an elimination half-life of approximately 12–20 h. While the precise temporal dynamics of pre- versus postsynaptic receptor engagement are not fully established in humans, lower doses are generally associated with relatively lower overall D2/D3 receptor occupancy. The timing of MRI acquisition in the present study was selected to coincide with the second plasma concentration peak, where central drug effects are expected to be relatively stable. On the day of the CBF assessment, study participants received a single dose of 100 mg amisulpride or matching placebo 3.5–4 h before entering the MR scanner. The scanning session consisted of an anatomical scan (5 min), a resting state scan (8 min), two fMRI tasks (25 min), and ended with the ASL sequence (10 min). The total scanning time was approximately one hour. The present report focuses on the CBF data. Analyses of the remaining imaging data will be reported elsewhere. To assess the plasma concentration of amisulpride, blood samples were taken at several time points: 30 min before drug administration, as well as 1 h, 3.5 h, and 4.5 h post-administration. The psychometric assessment was conducted on the day of the scanning session, and before participants entered the scanner. Adverse events (AEs) were assessed for safety at each study visit using a standardized protocol. A more detailed description of the experimental design and procedures is provided in a previous publication [52].

### Measures of depression and anhedonia

Clinical screening measures included the Structured Clinical Interview for DSM-5 (SCID-5 [53]), the Montgomery-Åsberg Depression Rating Scale [54], and the Columbia-Suicide Severity Rating Scale [55]. All interviews were conducted by trained professionals following standardized procedures. Subjects also completed the following self-report measures: Beck Depression Inventory (BDI-2 [56]), the Dimensional Anhedonia Rating Scale (DARS [57]), the Leuven Affect and Pleasure Scale (LAPS [58]), and the Snaith-Hamilton Pleasure Scale (SHAPS [59]). To improve effect sizes for brain-behavior relationships by increasing the reliability of self-report questionnaires, a confirmatory factor analysis was used to estimate the expression of anhedonia as a latent factor indicated by the SHAPS, DARS total, and LAPS hedonic tone subscale. By removing variance not attributable to the common latent factor (Anhedonia), this approach results in more accurate measures of the construct of interest and decreases the number of statistical tests conducted (see Supplementary Methods, Table S2, and [60]).

### Data acquisition and analysis

Acquisition of brain images was conducted using a 3 Tesla MRI scanner (PRISMA, Siemens Medical Systems, Erlangen, Germany) with a 20-channel head coil at the Berlin Center for Advanced Neuroimaging (BCAN). An anatomical brain image was acquired with a 3D T1-weighted scan (Magnetization Prepared Rapid Acquisition Gradient Echo sequence, TE = 3.03 ms, TR = 2.3 s, 192 slices, and FOV=256 × 256 × 192 mm). ASL data was acquired using a multi-post labeling delay pseudo continuous [61] ASL sequence (labeling duration = 1800 ms, post labeling delays = 400,700,1000,1300,1600,1900,2200, and 2500 ms, labeling plane positioned 90 mm below the center of the imaging region, background suppression using pre-saturation and two inversion pulses timed as per Günther et al. [62] with T1_opt_ = 700 ms, with nulling occurring 100 ms prior to excitation) with a segmented 3D-GRASE readout (4 segments, resolution = 3.4 × 3.4 × 3.3 mm, 38 slices, TR = 5 s, TE = 35 ms, 120° refocusing flip angle, left-right phase-encoding, 6/8 slice partial Fourier, 1 label/control pair per post labeling delay). Two separate calibration (M0) scans were also acquired with no ASL labeling or background suppression pulses using identical readout parameters: one with left-right and one with right-left phase-encoding to allow for B0-induced distortion correction. Total ASL scan time was 6 min 8 s. The ASL data were preprocessed using FSL tools, correcting for motion [63], B0-distortion [64], and coil sensitivity non-uniformity (by comparing the calibration images with and without pre-scan normalize corrections) before taking the control-label difference at each post labeling delay. Kinetic model fitting [65], accounting for a macrovascular component [66], was performed using a spatial Bayesian prior to stabilize the fitting process [67, 68]. Finally, relative perfusion (rCBF) was calculated relative to global CBF, with the added constraint that global CBF was restricted to a gray matter mask. Analysis of rCBF allows to further isolate regional changes in CBF from global CBF changes. From the preprocessed ASL images, mean CBF scores were extracted from the bilateral ventral striatum as primary endpoint. For exploratory analysis, mean CBF scores were extracted from the following nine ROIs: bilateral anterior and posterior insula, bilateral IFG, VTA, pallidum, caudate, putamen, dorsal ACC, and vmPFC. The ROIs were anatomically defined based on the Harvard-Oxford Atlas (thresholded at 25% probability), with the exceptions of the VTA and dorsal ACC (dACC), which were spherically defined based on automated term-based meta-analyses performed on neurosynth.org. The dACC ROI (radius = 10 mm) was centered at MNI coordinates [0, 32, 20]. Bilateral VTA ROIs (radius = 8 mm) were centered at [−8, −17, −18] and [8, −17, −18], respectively.

### Statistical analyses

A two‑way univariate ANOVA with the main factor group (MDD vs. HV) and the main factor treatment (placebo vs. amisulpride) was performed on the rCBF data of the bilateral ventral striatum, which was defined a priori as the primary endpoint of the study. In the case of a significant ANOVA effect (*p* < 0.05), Tukey’s post hoc tests were performed.

The same statistical procedures were applied to a small set of additionally examined, anatomically defined ROIs, which were considered exploratory. In addition, Pearson correlation analyses were conducted between rCBF, anhedonia scores, depression severity, and amisulpride plasma concentration. No correction for multiple comparisons across exploratory ROIs or for correlation analyses was applied, and these results should be interpreted as hypothesis‑generating. All statistical ROI analyses were conducted using JASP (Version 0.19.3).

Exploratory whole‑brain analyses were performed using unpaired t‑tests across the four treatment groups implemented in FSL FEAT. Whole‑brain results were thresholded at *p *< 0.05 family‑wise error corrected, with an initial cluster‑forming threshold of z > 3.1. Given the exploratory nature of these analyses, no further correction for multiple comparisons was applied.

## Results

*N* = 120 participants completed the study procedures (60 HV and 60 MDD patients). Gender ratio was 64:56 (F:M), mean age was 31.69 years (SD = 6.48). For an overview of participants’ baseline characteristics see Table 1. Nine additional participants had to be excluded due to insufficient ASL data quality. Four participants were excluded due to severe motion artifact. The others were excluded due to large changes in slice prescriptions between the different ASL sub-scans or labeling failures. The final sample for the analysis of the rCBF data consisted of 111 male and female participants (age: *M* = 31.48, *SD* = 6.36). Gender ratio was 59:52 (F:M). The final group sizes for the four treatment groups were as follows: HV-Amisulpride (HA, *n* = 25), HV-Placebo (HP, *n* = 29), MDD-amisulpride (PA, *n* = 27), MDD-placebo (PP, *n* = 30).

**Table 1:** Demographic, psychometric, and pharmacokinetic data presented by group.

		Group				Statistics		
		HA	HP	PA	PP	*p*(Group)	*p*(Treatment)	*p*(Interaction)
*N*		25	29	27	30			
Age	M	30.360	30.793	32.370	32.267	0.15	0.89	0.83
	SD	7.404	6.120	6.313	5.747			
Gender	F / M	14 / 11	13 / 16	16 / 11	16 / 14	0.52	0.37	na
MADRS	M	na	na	15.185	15.100	na	0.95	na
	SD	na	na	4.891	5.689			
BDI	M	0.960	1.172	20.111	16.233	<0.001	0.19	0.14
	SD	1.620	1.605	10.606	9.540			
SHAPS	M	0.520	0.172	3.963	3.200	<0.001	0.23	0.65
	SD	1.159	0.602	2.993	3.458			
DARS	M	60.320	59.621	42.037	43.000	<0.001	0.95	0.67
	SD	5.670	6.287	11.103	14.825			
LAPS	M	33.120	34.103	23.444	25.233	<0.001	0.29	0.76
	SD	5.563	4.229	8.178	8.369			
Anhedonia	M	-0.567	-0.653	0.677	0.495	<0.001	0.27	0.69
	SD	0.428	0.334	0.667	0.900			
C_p_ T1	M	86.920	na	67.963	na	0.15	na	na
	SD	56.149	na	36.723	na			
C_p_ T2	M	181.960	na	170.333	na	0.57	na	na
	SD	77.246	na	68.938	na			
C_p_ T3	M	137.880	na	126.259	na	0.41	na	na
	SD	56.560	na	44.721	na			

HA = healthy volunteers / amisulpride, HP = healthy volunteers / placebo, PA = patients / amisulpride, PP = patients / placebo, MADRS = Montgomery-Åsberg Depression Rating Scale, BDI = Beck Depression Inventory, SHAPS = Snaith-Hamilton-Pleasure-Scale, DARS = Dimensional Anhedonia Rating Scale, LAPS = Leuven Affect and Pleasure Scale, Anhedonia = latent variable derived from confirmatory factor analysis, C_p_ = Plasma Concentration, T1 = 1 h post-dose, T2 = 3.5 h post-dose, T3 = 4.5 h post-dose, na = not applicable.

*p*(Group), *p*(Treatment), and *p*(Interaction) refer to main effects and interactions from 2×2 ANOVAs with factors Group (HV vs. MDD) and Treatment (amisulpride vs. placebo), where applicable. When only two groups were compared, two‑sample *t* tests were used. Gender differences assessed using Chi‑square tests. MADRS analyses restricted to MDD participants. Pharmacokinetic analyses restricted to amisulpride‑treated participants.

**Fig. 1:**
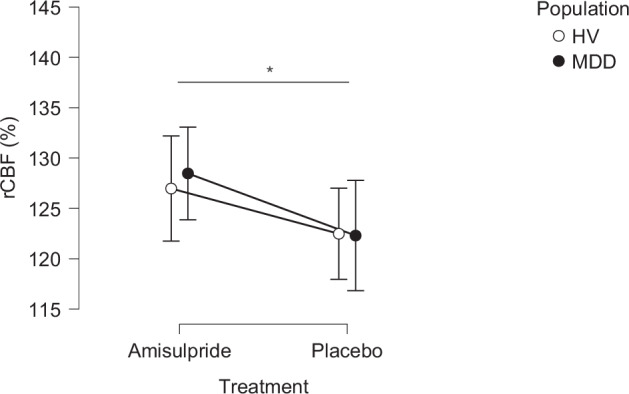
Relative cerebral blood flow (rCBF) in the bilateral ventral striatum. rCBF is shown as a function of diagnosis (major depressive disorder [MDD] vs. healthy volunteers [HV]) and treatment (amisulpride vs. placebo). rCBF values are expressed as percentages and represent regional cerebral blood flow normalized to global gray matter perfusion. Points represent group means; error bars indicate 95% confidence intervals. *p < 0.05.

The two-way ANOVA conducted for the bilateral ventral striatum revealed a significant main effect of treatment, F(1, 107) = 4.78, *p* = 0.031, η² = 0.043. Participants that received amisulpride (M = 127.72, SE = 1.78) had higher relative perfusion compared to participants that received placebo (M = 122.39, SE = 1.67, see Fig. 1). No significant main effect of group, and no significant interaction was observed. None of the additionally investigated ROIs showed significant main—or interaction effects (descriptive statistics for all ROIs are provided in the Supplementary Table S1). Furthermore, exploratory analyses of absolute (non-normalized) CBF revealed no significant effects of treatment, diagnostic group, or their interaction across the investigated regions of interest (all p > .05).

**Fig. 2:**
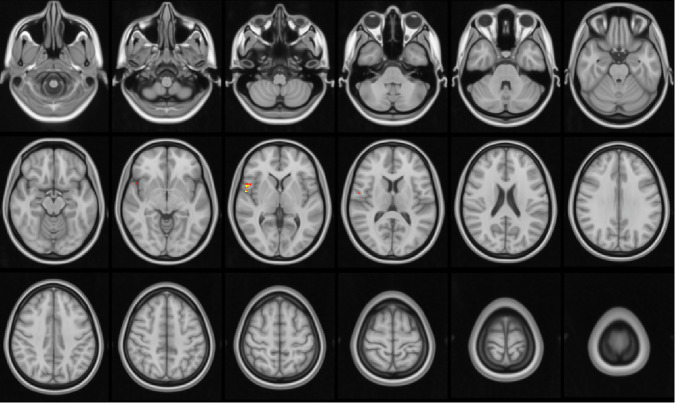
Whole-brain analysis of relative cerebral blood flow: MDD vs. healthy volunteers under placebo. Exploratory voxel-wise comparison (MDD/placebo vs. HV/placebo) revealed significantly higher perfusion in the right frontal operculum in MDD patients. The colored region depicts the significant cluster (103 voxels) surviving correction for multiple comparisons (*p* < 0.05, FWE-corrected). MNI coordinates of the peak voxel: [56, 0, 4]. Image is shown in radiological convention (left = right).

**Fig. 3:**
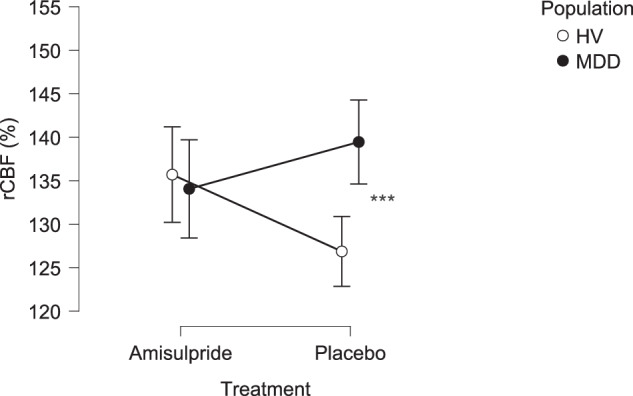
Relative cerebral blood flow (rCBF) in the right frontal operculum. rCBF is shown by diagnostic group (major depressive disorder [MDD] vs. healthy volunteers [HV]) and treatment (amisulpride vs. placebo). rCBF values are expressed as percentages and represent regional cerebral blood flow normalized to global gray matter perfusion. Points represent group means; error bars indicate 95% confidence intervals. ***p < 0.001.

The exploratory whole-brain analyses were conducted for the following pairwise comparisons: PA vs PP, HA vs HP, PA vs HA, and PP vs HP. It revealed one significant cluster in the right frontal operculum (MNI coordinates [56, 0, 4], cluster size = 103 voxels, *p* < 0.05, FWE corrected) for the HP vs. PP contrast (see Fig. 2). Subsequent ROI‑based analyses using mean rCBF values extracted from an anatomically defined right frontal operculum region (Harvard–Oxford cortical atlas) demonstrated a large effect size for this group difference (Cohen’s d = 1.07) that was not present between the groups that received amisupride (see Fig. 3). No other significant clusters were observed in the whole-brain analyses.

Correlation analyses revealed a significant positive relationship between anhedonia and rCBF in the ventral striatum in HV that received amisulpride (*r*(23) = 0.59, *p* = 0.002). This effect was not present in MDD and in participants that received placebo (all *p* > 0.2). *Fisher’s z*-test showed that the correlation in the HA group was significantly stronger compared to the PA group (*Z* = 2.92, *p* = 0.0036). Furthermore, a positive association was observed between anhedonia and rCBF in the right frontal operculum for subjects that received placebo (*r*(57) = 0.47, *p* < 0.001). This effect was not present in subjects that received amisulpride (*r*(50) = –0.017, *p* = 0.91). *Fisher’s z*-test showed that the correlation in the placebo group was significantly stronger compared to the amisulpride group (*Z* = 2.69, *p* = 0.007). A positive association was also observed between BDI scores and rCBF in the right frontal operculum for subjects receiving placebo (*r*(57) = 0.47, *p* < 0.001), but not in those receiving amisulpride (*r*(50) = –0.023, *p* = 0.87). *Fisher’s z*-test showed that the correlation in the placebo group was significantly stronger compared to the amisulpride group (*Z* = 2.72, *p* = 0.006). No other significant correlations were observed.

A total of 34 treatment-emergent adverse events (TEAEs) were reported by 26 participants. No serious or severe adverse events occurred, and no deaths were reported. TEAEs assessed as related to the study drug occurred across all treatment arms: 24 events weresince reported by 12 participants in the MDD/amisulpride group, 16 events by 10 participants in the MDD/placebo group, 8 events by 6 participants in the HV/placebo group, and 5 events by 5 participants in the HV/amisulpride group. Most events were mild and transient, with headache being the most frequently reported symptom.

## Discussion

To our knowledge, this is the first study to investigate the effects of a transient increase in dopamine signaling through amisulpride administration on CBF in a large sample of healthy subjects and patients with MDD. Importantly, the present study was designed to probe acute neurophysiological effects on reward-related circuits rather than to assess clinical antidepressant efficacy. We here focused on regions of the reward network and our results show higher perfusion in ventral striatum in both healthy and depressed subjects who were administered amisulpride. There were no effects of amisulpride on CBF in any other region of the reward network. Perfusion in the ventral striatum was associated with anhedonia. Assessed adverse events were mild and did not interfere with data acquisition or analysis. It is important to note that the use of a single low dose of amisulpride in the present study served as a pharmacological probe to transiently modulate dopaminergic signaling. In contrast, clinically meaningful antidepressant effects of amisulpride have been reported only following repeated administration. Therefore, the current findings should be interpreted as providing mechanistic insight into dopaminergic influences on reward-related neural activity, rather than as evidence of therapeutic efficacy.

A previous study investigating the effects of increased dopamine signaling on CBF in healthy subjects showed that continued administration of low-dose amisulpride (200 mg/day for 1 week) was associated not only with perfusion increases in the basal ganglia, but also with decreases in the cerebral cortex [50]. Our findings highlight that effects occur after a single administration of a lower dose of amisulpride (100 mg). At a molecular and cellular level, several mechanisms may account for the observed higher ventral striatal perfusion associated with amisulpride administration. One possibility is that low-dose amisulpride modulates presynaptic D2/D3 autoreceptors, reducing inhibitory feedback and thereby increasing dopamine release. Elevated extracellular dopamine may in turn enhance activation of postsynaptic D1 receptors, leading to increased neuronal activity. Through neurovascular coupling, this increased metabolic demand may manifest as higher regional cerebral blood flow. Alternatively, postsynaptic D2/D3 receptor blockade may also contribute to altered striatal signaling dynamics. These mechanisms are not mutually exclusive, and their relative contributions likely depend on dose, receptor distribution, and pharmacokinetic factors. Importantly, the present data do not allow these mechanisms to be disentangled and should therefore be interpreted cautiously.

Consistent with this mechanistic framework, studies that probed neural activity using fMRI reported increased ventral striatum activity during reward processing in MDD patients, but not in healthy subjects after a single low dose (50–200 mg) of amisulpride [14, 21, 22, 69]. Striatal perfusion did not differ between placebo- treated healthy and depressed subjects, contradicting some previous studies that reported higher striatal CBF in MDD. Higher striatal perfusion in MDD has been proposed as a marker of impaired mood regulation [36, 39, 42, 45]. However, blunted striatal reactivity might be specific for MDD patients with impaired mood reactivity [70]. Ventral striatum hypofunction has been implicated in both depression and negative symptoms of schizophrenia and it has been suggested that higher CBF might be related to enhanced responsiveness and thereby be relevant to mechanisms of therapeutic benefit [71]. Since self-reported anhedonia was linked to higher CBF in the ventral striatum among subjects who received amisulpride, it could be hypothesized that the increase in striatal perfusion potentially reflecting dopaminergic signaling is more pronounced in individuals with reward processing impairments. This, in turn, suggests that those with greater deficits in reward functioning may experience greater benefits from the antidepressant properties of amisulpride, although no conclusions about clinical antidepressant effects can be drawn from the present single-dose design. However, the relationship between anhedonia and increased striatal perfusion was not observed in individuals with MDD. This may be due to the single administration of amisulpride, as longer or more sustained exposure could be necessary for associations between symptoms and neural function to become apparent in MDD. Supporting this idea, a previous study by Admon et al. [14] showed enhanced striatal function, but no effect on behavior (reward learning) after a single low dose of amisulpride. Also, antidepressant effects of amisulpride in depressed individuals have been reported following prolonged, but not acute, administration [72].

Exploratory whole-brain analysis in the placebo-treated subjects revealed significantly greater CBF of the right frontal operculum in MDD patients compared to healthy controls, whereas this group difference was not observed when subjects received amisulpride. Accordingly, both anhedonia and depression severity were associated with CBF in the right frontal operculum in subjects receiving placebo, but not in those receiving amisulpride. Importantly, the frontal operculum finding emerged from exploratory whole‑brain analyses and was identified without additional correction across contrasts. Therefore, this result should be interpreted as hypothesis‑generating rather than confirmatory, and requires independent replication.

The frontal operculum covers parts of the ventral frontal cortex adjacent to the insula and is involved in language, somatosensory, and cognitive functions [73, 74]. Its network connections involve both proximal structures, such as the insula, and remote processing areas, such as the ACC, and it has been proposed as a key node for exerting control over cognitive processes [75, 76]. The ACC-operculum connection belongs to the salience network [77, 78], and a previous meta-analysis reported that the frontal operculum is also part of the reward network and recruited primarily during social reward and attention processing [79]. Accordingly, abnormal opercular responses during reward processing have been linked to anhedonia in schizophrenia [80]. While findings on CBF abnormalities in MDD have been inconsistent in terms of localization and directionality [33, 45], several previous studies have reported higher perfusion in insula and frontal cortical areas, which was associated with depression severity and decreased with response to antidepressant treatment [38, 41, 42]. Since there were no differences in operculum perfusion between healthy subjects and MDD patients after administration of amisulpride, one might hypothesize that an increase in dopamine signaling might have a localized effect on CBF that is in turn relevant for depression severity, specifically for anhedonia. Impaired coordination among large-scale brain circuits, including corticostriatal pathways, has been proposed as a key factor in the pathophysiology of depression [81]. The interaction between dopamine-rich areas of striatum and cortical regions may be crucial for reward processing, and dopamine enhancement may help to regulate this functional circuit in depression. Supporting this idea, a previous study demonstrated potentiated corticostriatal functional connectivity after amisulpride administration in MDD patients, but not in healthy subjects [14]. Accordingly, our results show that amisulpride seems to have differential effects on operculum perfusion in healthy and MDD subjects. In healthy subjects, the placebo group showed lower CBF than the amisulpride group, while in MDD patients, CBF was higher in the placebo group. Results of our whole-brain analyses showed no extensive effects of amisulpride which rather argues against the possibility of a general and direct effect on blood vessels. Underscoring this point, perfusion in the investigated regions was not associated with plasma amisulpride concentration.

We here examined a large sample of unmedicated MDD patients and healthy controls using a double-blind, placebo-controlled design. However, there are several limitations to the study. Previous work has shown that the plasma concentration of amisulpride has two peaks, at approximately 1 h, and between 3 and 4 h after administration [82]. Here, the ASL measurement occurred approximately 4 h after amisulpride administration, and our pharmacokinetic data showed a decrease in plasma concentration between 3.5 and 4.5 h after administration. While our results clearly demonstrate effects of amisulpride on rCBF in ventral striatum and frontal operculum, an earlier measurement might have detected perfusion changes in further regions of the reward network. Although low-dose amisulpride is thought to increase dopaminergic signaling via presynaptic autoreceptor blockade, the present study did not directly measure dopamine transmission. ASL-derived rCBF reflects neurovascular coupling and provides an indirect index of neural activity, which may be influenced by multiple neurochemical systems. Therefore, the observed perfusion changes should be interpreted as being consistent with, but not definitive evidence of, dopaminergic modulation. An additional limitation relates to the between-subject design of the study. As each participant was scanned only once following drug administration, it is not possible to determine whether the observed differences reflect an absolute increase in perfusion following amisulpride or a decrease in the placebo condition. Therefore, the results should be interpreted as indicating relative differences between treatment groups rather than within-subject changes. The majority of patients had only mild MDD, which may explain why we could not confirm previously reported differences in perfusion between patients and healthy controls. However, supporting the validity of our findings, patients and controls differed significantly in self- reported anhedonia, which, together with depression severity, was significantly associated with increased perfusion in the frontal operculum. Furthermore, the single-dose design limits the ability to draw conclusions about clinical antidepressant mechanisms. While low-dose amisulpride is known to exert antidepressant effects with repeated administration, the present study captured only acute neurophysiological responses. As such, the observed CBF changes should be interpreted as reflecting transient pharmacological modulation of neural circuits rather than treatment-related changes. We here administered a single amisulpride dose of 100 mg, whereas previous studies used either 50 mg [14] or 200 mg [21, 22]. Antidepressant effects have been reported for low doses ≤200 mg [17–19, 72], and both the pharmacokinetic data and the region-specific effects on perfusion found here suggest that administration of 100 mg resulted in enhanced dopamine signaling. Although sex distribution was balanced across experimental groups, the study was not powered to investigate sex- or gender-specific effects or interactions with treatment.

In conclusion, we present the first evidence of region-specific effects of increased dopamine signaling through amisulpride administration on cerebral blood flow (CBF). Higher anhedonia-related CBF was observed in the ventral striatum but not in other regions of the reward network in both healthy and depressed individuals administered with amisulpride. CBF in the right frontal operculum was also linked to anhedonia and was higher in MDD patients compared to healthy individuals, a difference that was eliminated following amisulpride administration. These findings suggest that modulation of dopamine transmission may selectively influence corticostriatal regions involved in reward processing and anhedonia.

## Supplementary information

Supplement

## Data Availability

The individual de-identified participant data and the statistical code can be accessed upon reasonable request from the corresponding author.
