# Supplementary material for: Investigating the effect of increased dopamine signaling on cerebral blood flow in Major Depressive Disorder: a double-blind randomized placebo-controlled study
Source: Neuropsychopharmacology. 2026 Jun 26;51(10):1808–15. doi: 10.1038/s41386-026-02471-6 (PMC13486671; doi:10.1038/s41386-026-02471-6)
Supplement: Supplementary file 1 [file 41386_2026_2471_MOESM1_ESM.docx]

**Supplementary Material**

**Supplementary Methods**

CFA – Anhedonia factor scores

First, indicators were z-standardized to ensure similar scaling of the observed variables. To test for multivariate normality, the energy test was used as implemented in the MVN package (Korkmaz et al., 2014). Subsequently, the R package lavaan was employed to fit the model and extract model fit indices, factor loadings and their associated 95% bootstrap confidence intervals as well as anhedonia factor scores on the single subject level (Rosseel, 2012). Since only three indicators were used, the unstandardized factor loading of the SHAPS was fixed to one to overidentify the model and enable calculation of model fit indices. A maximum likelihood estimator was used to fit the model, assuming CFI ≥ .95, TLI ≥ .9 and SRMR ≤ .08 to indicate good fit (Byrne, 1994; Hu & Bentler, 1999). RMSEA will be reported for descriptive reasons but not interpreted due to its known unreliability in face of small degree of freedoms and small sample sizes (Kenny et al., 2015).

Results:

A latent factor model was fitted using confirmatory factor analysis including self-report data from 120 subjects. Energy and Mardia tests suggested violation of the multivariate normality assumption (Energy: *E-statistic* = 2.426, p < .001). The measurement model included three indicators (SHAPS, DARS total scores and LAPS hedonic tone subscale) and a single higher-order factor (anhedonia). Overall, model fit indices provided evidence for good to excellent model fit (*χ^2^*(1) = 1.933, *p_boot_* = .096, *CFI* = .996, *TLI* = .989, *SRMR* = .150, *RMSEA* = 0.088) and high factor loadings were observed (see table S1).

Adverse Events

A total of 34 treatment-emergent adverse events (TEAEs) were reported by 26 participants. No serious or severe adverse events occurred, and no deaths were reported. TEAEs assessed as related to the study drug occurred across all treatment arms: 24 events were reported by 12 participants in the MDD/amisulpride group, 16 events by 10 participants in the MDD/placebo group, 8 events by 6 participants in the HV/placebo group, and 5 events by 5 participants in the HV/amisulpride group. Most events were mild and transient, with headache being the most frequently reported symptom.

**Supplementary Tables**

**Table S1.** CFA factor loadings

| Scale | *Unstandardized factor loading* | *λ* | *95%-BCI* | *z* |
| --- | --- | --- | --- | --- |
| SHAPS | 1 | .919 | - | - |
| LAPS hedonic tone subscale | -0.887 | -.845 | [-1.023, -0.775] | -14.004 |
| DARS total score | -0.983 | -.926 | [-1.136, -0.851] | -13.441 |

Note: 95%-BCI reflect bootstrap confidence intervals for the unstandardized factor loadings. BCI and z values for the SHAPS are not provided since its factor loading was fixed to one to overidentify the model and enable calculation of model fit indices. λ = standardized factor loadings; SHAPS = Snaith Hamilton Pleasure Scale, DARS = Dimensional Anhedonia Rating Scale, LAPS = Leuven Affect and Pleasure Scale, NA = not applicable.

**Table S2**. Descriptive statistics of mean relative perfusion (M±SD) in the investigated bilateral ROIs presented by group.

| Bilateral ROIs | HA | HP | PA | PP |
| --- | --- | --- | --- | --- |
| ventral striatum | 126.97 (12.65) | 122.48 (11.9) | 128.47 (11.65) | 122.3 (14.67) |
| anterior insula | 128.65 (17.49) | 128.62 (9.78) | 127.61 (10.35) | 130.87 (11.2) |
| posterior insula | 127.48 (17.69) | 132.25 (14.32) | 125.07 (12.16) | 129.84 (13.7) |
| inferior frontal gyrus | 103.29 (10.64) | 99.9 (8.25) | 101.21 (8.35) | 102.29 (9.39) |
| VTA | 82.85 (15.52) | 81.83 (10.67) | 85.13 (14.05) | 87.62 (13.65) |
| pallidum | 80.79 (11.19) | 75.96 (8.15) | 81.32 (9.36) | 79.28 (11.44) |
| caudate | 100.79 (13.85) | 97.49 (13.49) | 98.64 (14.13) | 94.57 (11.15) |
| putamen | 114.77 (12.95) | 110.51 (8.02) | 111.78 (8.56) | 109.87 (11.2) |
| dorsal ACC | 111.23 (11.73) | 108.58 (9.49) | 112.04 (12.92) | 109.75 (11.64) |
| ventromedial PFC | 117.34 (11.36) | 118.04 (10.7) | 119.54 (9.72) | 117.94 (13.76) |
| frontal operculum (right) | 135.7 (13.3) | 126.87 (10.55) | 134.06 (14.28) | 139.45 (12.92) |

Note: Values represent mean (M) and standard deviation (SD). HA = healthy volunteers with amisulpride; HP = healthy volunteers with placebo; PA = patients with amisulpride; PP = patients with placebo. ROI = region of interest; VTA = ventral tegmental area; ACC = anterior cingulate cortex; PFC = prefrontal cortex.

**Supplementary Figures**

**Figure S1.** Participant flowchart illustrating screening, exclusion, randomization, group allocation, and dropout across study arms.

Note: Of 401 individuals screened, 184 patients with major depressive disorder (MDD) and 217 healthy volunteers (HV) were identified. After exclusions, 127 participants were randomized to one of four groups (MDD/placebo, MDD/amisulpride, HV/placebo, HV/amisulpride). A total of 120 participants completed all study procedures and were included in the final analysis. Reasons for exclusion and dropout are detailed in the respective boxes.
